# Approach to acute kidney injury in HIV-infected patients in South Africa

**DOI:** 10.4102/sajhivmed.v18i1.714

**Published:** 2017-11-28

**Authors:** Michael T. Boswell, Theresa M. Rossouw

**Affiliations:** 1 Department of Medical Immunology, University of Pretoria, South Africa; 2 Nuffield Department of Medicine, University of Oxford, United Kingdom

## Abstract

**Background:**

HIV-infected patients have an increased risk of renal disease. Current first-line antiretroviral therapy contains tenofovir disoproxil fumarate (TDF), which has nephrotoxic potential, characterised by proximal tubular cell injury. This may result in acute kidney injury, chronic kidney disease or partial or complete Fanconi syndrome.

**Objectives:**

We reviewed the existing literature on acute kidney injury and TDF-associated nephrotoxicity with the aim of providing an approach to diagnosis and management, which is relevant to a general medical practitioner.

**Methods:**

We performed a broad literature search of biomedical databases including PubMed and ScienceDirect. Our search terms included, but were not limited to, ‘tenofovir’, ‘nephrotoxicity’, ‘HIV’, ‘acute kidney injury’ and ‘renal tubular acidosis’.

Our aim was not to generate a systematic literature review with weighted evidence, but rather to provide a review of best practice from a variety of sources. Where published studies were not available from the above databases, we relied on relevant textbooks and professional guidelines.

**Results:**

Potential nephrotoxicity is not an impediment to the widespread use of TDF in treating HIV infection, because most patients will tolerate the medication well. However, patients with advanced disease, low body weight, advanced age, pre-existing kidney disease and concomitant use of other nephrotoxic medications are at increased risk of adverse renal events and may develop severe complications if not appropriately managed. These risk factors are unfortunately common in patients initiating antiretroviral therapy in South Africa.

**Conclusion:**

Prevention of renal damage by means of careful screening and monitoring of high-risk patients is of paramount importance. Increased awareness of this problem and knowledge of how to manage kidney disease should be emphasised for general medical practitioners who work with HIV-infected patients.

## Introduction

HIV is South Africa’s leading health problem. Approximately 7 million people are thought to be HIV-infected and the prevalence is increasing because of improved life expectancy on combination antiretroviral therapy (ART).^1^ Current estimates suggest that approximately 3.4 million South Africans are receiving ART, and this number is expected to increase dramatically with the country adopting the 2015 World Health Organization (WHO) guidelines recommending that all HIV-infected patients receive ART regardless of CD4 count. Because of constraints on resources, the options available for ART in the public health services are relatively limited. The National Department of Health recommends a first-line regimen consisting of a nucleotide/nucleoside reverse transcriptase inhibitor (NtRTI/NRTI) backbone together with efavirenz, with the backbone consisting of a combination of tenofovir disoproxil fumarate (TDF) and lamivudine (3TC) or emtricitabine (FTC). One major concern regarding such widespread use of TDF is nephrotoxicity. This article gives an overview of the causes and management of renal dysfunction in HIV-infected patients, with special emphasis on acute kidney injury (AKI) and TDF-associated nephrotoxicity. Our aim is to provide primary healthcare practitioners with a working system for managing AKI in the context of HIV.

## Acute kidney injury and chronic kidney disease

HIV infection is associated with an almost four-fold increased risk of renal disease, attributable to a variety of causes of AKI as well as chronic kidney disease (CKD).^2^ The most common causes are summarised in Table 1.^11^ AKI can be caused by pre-renal (haemodynamic alterations), renal (nephritis, nephrosis, tubulopathies and necrosis) and post-renal (crystal nephropathy) factors. In HIV-infected patients, acute tubular necrosis (ATN) secondary to hypotension or sepsis is the most common cause of AKI, with WHO stage IV disease, low CD4 counts and hypoalbuminaemia all associated with higher mortality.^3^ Many commonly used medications have nephrotoxic potential (Table 2)^12^, varying from predictable, cumulative dose-dependent nephrotoxicity to idiosyncratic dose-independent toxicity, or both.^4^

**TABLE 1:** Common causes of acute kidney injury and chronic kidney disease in HIV-infected patients.

Acute kidney injury	Chronic kidney disease
Dehydration secondary to gastroenteritis	HIV-associated nephropathy with focal glomerulosclerosis, or classic HIV-associated nephropathy
Opportunistic infections and sepsis, not necessarily with documented hypotension	HIV-immune complex deposition, often associated with hepatitis B/C co-infection
Nephrotoxic medication†; drug-induced interstitial nephritis	Various glomerulonephropathies such as amyloidosis
HIV-associated thrombotic thrombocytopenic purpura – haemolytic uraemic syndrome	Comorbid diseases such as hypertension or diabetes mellitus

† See Table 2.

**TABLE 2:** Medication with nephrotoxic potential.

Drug class	Subclass	Example
Nonsteroidal anti-inflammatory drugs (NSAIDs)	Nonselective NSAIDs	Diclofenac Naproxen Ibuprofen Indomethacin
	Cyclooxygenase-2 (COX-2)-specific NSAIDs	Celecoxib Rofecoxib
Antihypertensives	ACE inhibitors (ACEI)	Perindopril
	Angiotensin II receptor blockers (ARBs)	Losartan
	Diuretics	Loop diuretics, e.g. furosemide Thiazides Triamterene
Antimicrobials	Aminoglycosides	Neomycin (most toxic) Gentamicin Tobramycin Amikacin Streptomycin (least toxic)
	Sulfa-based antibiotics	Sulfamethoxazole–trimethoprim Sulfadiazine
	Glycopeptides	Vancomycin
	Fluoroquinolones	Ciprofloxacin
Antimycobacterials	Drug-susceptible tuberculosis	Isoniazid Ethambutol Rifampicin
	Drug-resistant tuberculosis	Capreomycin
Antivirals	-	Acyclovir Ganciclovir
	-	Foscarnet
	Antiretrovirals	Tenofovir Ritonavir†
Antifungals	-	Amphotericin B
Chemotherapeutics	-	Cisplatin Ifosfamide Methotrexate
	Anti-angiogenic drugs	Monoclonal antibodies against vascular endothelial growth factor Antagonists of vascular endothelial growth factor (VEGF) receptor
	Bisphosphonates	Intravenous pamidronate Intravenous zoledronate
Immunosuppressive agents	-	Tacrolimus Cyclosporine
Osmotic agents	-	Intravenous immune globulin Hydroxyethyl starch Mannitol Radiocontrast media
Mood stabilisers	-	Lithium Haloperidol
Analgesics	-	Paracetamol Acetylsalicylic acid
Proton-pump inhibitors	-	Lansoprazole Omeprazole Pantoprazole
HMG-CoA reductase inhibitors (statins)	-	Simvastatin
Herbal medication	-	Aristolochia fangchi
Xanthine oxidase inhibitor	-	Allopurinol
Anticonvulsant	-	Phenytoin

† Shafi et al.^13^

VEGF, vascular endothelial growth factor; HMG-CoA, 3-hydroxy-3-methylglutaryl-coenzyme A; ACE, angiotensin converting enzyme.

Acute kidney injury (AKI) is defined as an increase in serum creatinine by 26.5 mmol/L within 48 h; or an increase in serum creatinine to 1.5 times baseline, which is known or presumed to have occurred within the prior seven days; or a urine volume < 0.5 mL/kg/h for 6 h (this can, however, also occur with fluid restriction in the absence of AKI).^5^ Some studies have shown that complete recovery of renal function occurs in only 59% of HIV-infected patients presenting with AKI, with 2% of patients progressing to end-stage renal disease (ESRD).^3^ In practice, it may be difficult to distinguish AKI from CKD at presentation, because multiple biochemical abnormalities are common to both entities, including electrolyte abnormalities and proteinuria.

Chronic kidney disease is defined as abnormalities of kidney structure or function, present for at least three months, with implications for health. Classification is based on cause, glomerular filtration rate (GFR) and albuminuria category.^6^ AKI is a significant risk factor for the development of CKD and vice versa. A common feature is the disruption of normal renal architecture in the setting of a variety of adaptive immunological or vascular events, the interactions of which determine recovery or decline of renal function. A full discussion of the spectrum of CKD associated with HIV infection is beyond the scope of this article.

Both AKI and CKD are associated with cardiovascular disease, which has become a leading cause of morbidity and mortality in HIV-infected populations.^7^ HIV-infected patients are known to be at increased risk of cardiovascular disease because of increased prevalence of traditional risk factors (e.g. smoking, hypertension and dyslipidaemia), direct viral effects, chronic inflammation and ART use.^8^ TDF is especially important in this regard because it can decrease GFR and impair the activation of vitamin D in the renal proximal tubules, leading to vitamin D deficiency. Both decreased GFR and vitamin D deficiency have been associated with increased cardiovascular risk.^9,10^

## Tenofovir disoproxil fumarate-associated nephrotoxicity

The proximal tubular cells of the kidney are especially vulnerable to potential TDF toxicity because their unique cell membrane transporters promote entry of the drug into the cells.^14^ In addition, limited anaerobic adenosine triphosphate-generating capacity makes these cells vulnerable to mitochondrial dysfunction.^15^ Even though TDF is considered a weaker inhibitor of mitochondrial DNA polymerase γ than the NRTIs, the main pathophysiological mechanism underlying TDF nephrotoxicity appears to be mitochondrial damage.^16^ The proximal tubules are responsible for most of the tubular transport of molecules such as glucose, activation of 25-dihydroxycholecalciferol and release of ammonia necessary for proton secretion into the urine by distal segments. Mitochondrial damage will therefore negatively impact molecular transport and vitamin D activation, as well as urinary acidification.^14^ The resultant features include low serum levels of uric acid, phosphate and bicarbonate, together with high urine levels of glucose, low-molecular-weight proteins (e.g. β2-microglobulin), uric acid or phosphorus.^14^ The clinical spectrum of TDF-associated proximal tubular dysfunction is shown in Table 3.^17^

**TABLE 3:** Spectrum of tenofovir disoproxil fumarate-associated proximal tubular dysfunction.

Condition	Proximal renal tubular acidosis	Proximal tubule dysfunction	Fanconi syndrome
Serum pH	Acidotic	Acidotic	Acidotic
Serum biochemical abnormalities	Hypokalaemia results if bicarbonate therapy is instituted. Therefore, therapy relies on bicarbonate and potassium replacement.	Hypokalaemia, independent of bicarbonate replacement Hypophosphataemia	Hyphosphataemia Hypokalaemia
Urine abnormalities	-	Proteinuria	Hyperuricosuria Hyperphosphaturia Hypercalciuria Aminoaciduria Glycosuria
Effect on bone	-	-	Osteomalacia and rickets

Pharmacokinetic studies have shown a correlation between exposure to higher TDF concentrations and an increased risk of CKD over time. Two possible mechanisms contributing to increased TDF levels have been suggested: (1) polymorphisms in genes coding for cellular transport mechanisms may promote the accumulation of TDF in tubular cells and (2) decreased excretion brought about by low GFR may increase TDF plasma concentrations.^18,19^

TDF-induced renal toxicity may manifest as AKI, CKD or proximal tubular injury, including Fanconi syndrome, isolated hypophosphataemia and decreased bone mineral density.^20,21,22^ Proximal tubular dysfunction may result in an increase in serum creatinine in the absence of AKI because of a decrease in excretion of creatinine by this segment of the nephron.^23^ The risk for AKI on TDF therapy has been estimated to be approximately 1% in clinical trials and almost 2% in cohort studies. A recent meta-analysis found a significantly faster loss of kidney function in patients receiving TDF compared with controls.^24^ Most studies have, however, not found a significantly higher risk of proteinuria, CKD or ESRD requiring dialysis, and the above meta-analysis did not find an increased risk of hypophosphataemia, decreased bone mineral density or bone fractures in patients on TDF.^24,25,26,27^ The authors concluded that the overall risk was modest and it supported the routine use of TDF in settings where appropriate monitoring was available.

On a cautionary note, however, the meta-analysis mostly included patients from resource-rich countries with advanced medical systems and early initiation of ART. This is attested to by the fact that the median CD4 count was 364 cells/µL. In addition, the studies excluded patients with advanced disease and medical comorbidities and are therefore unlikely to accurately reflect the reality in sub-Saharan Africa, where late presentation and advanced disease at the time of ART initiation are still the norm.^28^ As a result of the complex pathologies present in advanced HIV, it is often very difficult to discern a single cause for AKI in many patients. A recent study in South Africa showed an incidence of renal impairment of a little below 3% during the first 12 months of TDF-containing ART in primary care populations,^29^ while another South African study of patients hospitalised with AKI reported TDF therapy to be associated with more rapid worsening of renal function, a higher likelihood of proteinuria and acidosis, and delayed renal recovery.^30^ The latter study highlights the poor prognosis of AKI in HIV-infected individuals in the context of limited access to renal replacement therapy, as more than a quarter of the patients in this cohort died.^30^

## Risk factors for tenofovir disoproxil fumarate-associated nephrotoxicity

The main risk factors for TDF nephrotoxicity include pre-existing renal impairment, older age, low body weight, advanced HIV disease (low CD4 count or AIDS), comorbidities (especially diabetes, hypertension and hepatitis C co-infection), concomitant use of nephrotoxic drugs and protease inhibitors.^14,23,31^ Countries in sub-Saharan Africa can expect to experience a large proportion of patients in whom the above-mentioned risk factors are present at ART initiation. A significant proportion of patients are underweight^28,32^ and a recent review of patients initiated on ART in South Africa between 2010 and 2014 reported a median CD4 count of only 213 cells/µL (interquartile range (IQR) 117–324 cells/µL).^33^ In addition, *Cryptococcus neoformans* is the leading cause of meningitis in South Africa, with the consequence that many patients will receive amphotericin B.^34^ The large number of patients co-infected with *Mycobacterium tuberculosis* may also contribute to increased risk because of rifampicin-related nephrotoxicity and interstitial nephritis induced by immune reconstitution inflammatory syndrome.^30^

Tenofovir disoproxil fumarate-associated nephrotoxicity generally, manifests within the first 3 to 9 months of treatment^35,36,37^ but a progressive decrease in eGFR has been demonstrated up to five years on ART, especially in patients with low body weight.^31^ Serum creatinine in the first four months of ART has a low predictive value for a change in eGFR after a year on ART^29^ and it is therefore essential that renal function in patients on TDF be monitored over the long-term. Questions remain about the ideal timing and tests to be used.

## Diagnosis of acute kidney injury

Early detection of nephrotoxicity and withdrawal of offending drugs are key to avoiding irreversible renal damage.^14^ It is, however, equally important to remember that TDF is not the only cause of renal disease and that failure to consider other causes may result in a missed opportunity for the diagnosis of a significant underlying condition requiring intervention.^38^

Initial evaluations, which may be used to determine whether TDF nephrotoxicity is present, include the following:

- serum urea, electrolytes, creatinine and eGFR
- serum calcium, magnesium and phosphate
- urine phosphate and urea (if possible, fractional excretion of phosphate should be done as it is a more accurate measurement of proximal tubular function)
- spot urine protein to creatinine ratio
- urine dipstick for glycosuria.

The aim of these investigations is to give an indication of the extent of renal damage, as well as to differentiate between glomerular and tubular dysfunctions. Glomerular dysfunction presents with proteinuria with or without haematuria and is usually an indication of HIV-related renal disease (e.g. HIV-associated nephropathy [HIVAN]) or other chronic diseases, such as diabetes mellitus. Drug toxicity commonly presents with tubular dysfunction characterised by glycosuria, hyperphosphaturia and hypophosphataemia. Isolated haematuria is often indicative of an extra-renal problem with pathology localised to the ureter, bladder or prostate.^38^

Because the effect of TDF on glomerular function is believed to be mild, measuring only eGFR and albuminuria is not an appropriate screening strategy and is unlikely to detect early TDF nephrotoxicity. The presence of tubular proteinuria is thought to be the most sensitive test for proximal tubule dysfunction, and a spot urine protein to creatinine ratio or the urinary retinol-binding protein to creatinine (RBP:Cr) ratio has been suggested as a useful marker and screening tool for TDF-associated tubular toxicity.^23,39^ Further validation of the latter test is needed, and until it is routinely available, measuring the spot urine protein to creatinine ratio or less sensitive, but well-established, markers of proximal tubule dysfunction, such as increased fractional excretion of phosphate and glycosuria, are the most appropriate alternatives.

**FIGURE 1:**
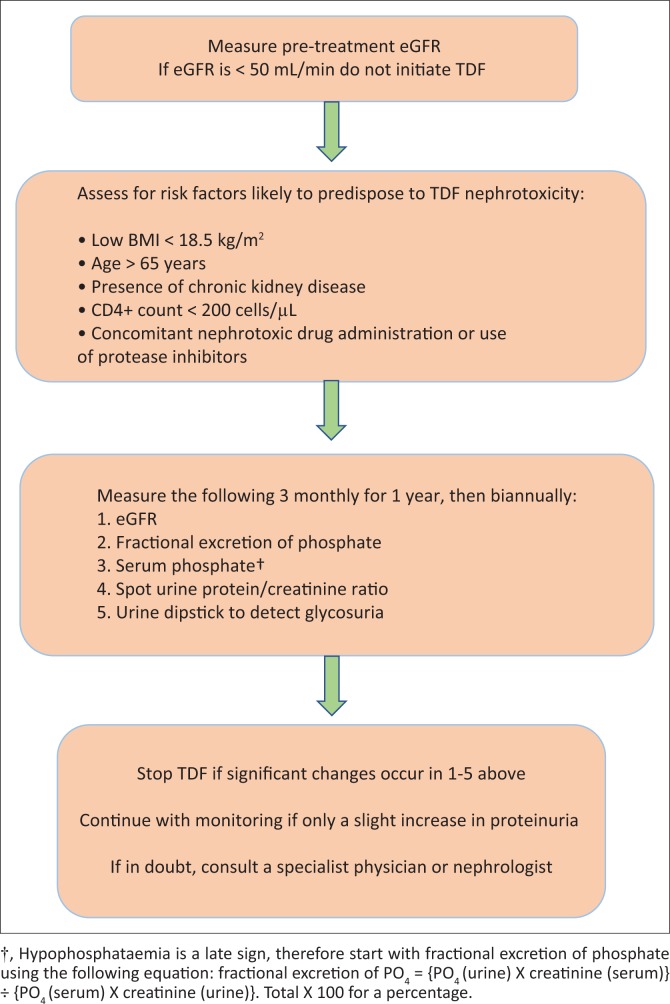
Algorithm for initiation of tenofovir disoproxil fumarate and monitoring of tenofovir disoproxil fumarate-associated nephrotoxicity. Should multiple risk factors for TDF toxicity be present in a patient, closer monitoring may be desirable.

All patients should have an eGFR calculated before initiation of any ART. Patients with an eGFR > 60 mL/min do not warrant further investigation and can be monitored routinely. Patients with the above risk factors should, however, receive closer monitoring for TDF nephrotoxicity, as demonstrated in the proposed algorithm in Figure 1.^23^ It should be kept in mind that eGFR calculated by means of the Modification of Diet in Renal Disease (MDRD) or Cockcroft–Gault formula may underestimate the degree of renal dysfunction if a patient’s muscle mass is lower than the age and sex standards,^40^ in which case a 24-h urine specimen for creatinine clearance calculation would be more appropriate. The CKD-Epi equation has been proposed as a replacement to the MDRD formula because of its greater accuracy in estimating GFR.^41^

## Management of acute kidney injury

**FIGURE 2:**
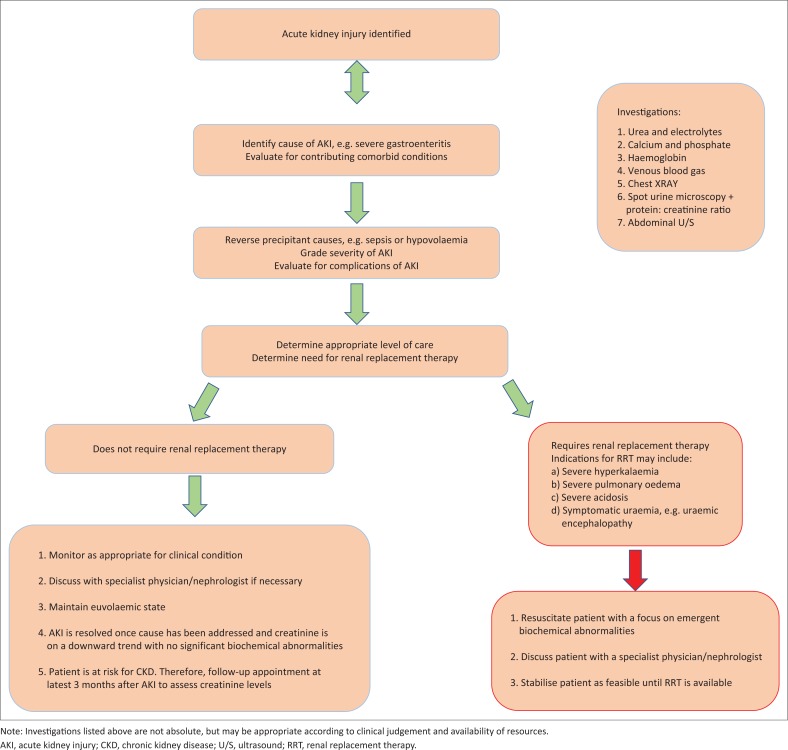
Algorithm for management of acute kidney injury, based on the KDIGO 2012 acute kidney injury guidelines.

The specific cause and clinical context of the patient determines the management of AKI. A general approach, based on the KDIGO 2012 AKI guidelines,^5^ is shown in Figure 2.^5^ Three common clinical scenarios can be foreseen, which are discussed below.

### Patients not yet on antiretroviral therapy who present with acute kidney injury

Patients with AKI should be evaluated promptly to determine the cause, with special attention to reversible causes, and the need for renal replacement therapy must be assessed. Hospitalisation will frequently be required and, where available, specialist referral is advised.^5^ Patients should be managed according to stage and cause and assessed after three months to determine if the AKI has resolved or progressed to CKD. In patients with pre-existing CKD, a 3-month follow-up is used to monitor for further deterioration in renal function. Initiation of ART should be deferred until AKI has resolved with creatinine clearly on a downward trend and an eGFR suitable for the selected ART.^42^ Should a patient have AIDS, CD4 count < 50 cells/µL or severe HIV-related disease such as thrombotic thrombocytopaenic purpura, initiation of ART may be the overriding therapeutic goal even if AKI has not yet resolved. In such cases, the patient should preferably be discussed with a specialist and ART be restricted to non-nephrotoxic drugs, for example, abacavir (ABC), provided that the pre-treatment viral load (VL) is below 100 000 copies/mL; zidovudine (AZT), provided that the patient is not anaemic; or, alternatively, as a short-term option, stavudine (d4T).^42^

### Patients who have been initiated on tenofovir disoproxil fumarate and now have acute kidney injury

Current local guidelines recommend that TDF be substituted when a patient’s eGFR is < 50 mL/min (calculated using either the Cockcroft–Gault formula or the MDRD formula).^42^ In patients with AKI, dosages of NRTIs and some other commonly used drugs should be adjusted based on eGFR (Table 4).^42,45,46^ TDF should be interrupted even if it is not thought to be the cause of AKI and substituted with an alternative NRTI. Once there is clear evidence that renal function is improving (creatinine on downward trend), NRTI dosages should be readjusted to standard dosages to avoid under-dosing.^42^ We recommend that additional screening should be undertaken to evaluate proximal tubule function and exclude renal Fanconi syndrome. In addition, if no reversible cause for AKI is identified, it is reasonable to assume that TDF is the cause and it should be either discontinued or monitoring increased as appropriate.

**TABLE 4:** Medication adjustments in acute kidney injury and/or chronic kidney disease.

Medication	eGFR 10–50 mL/min	eGFR < 10 mL/min	Additional points
Amikacin	12 mg/kg – 15 mg/kg 2 or 3 times per week	-	Better tolerated than kanamycin, but more expensive
Amphotericin B	No specific eGFR cut-off. If creatinine doubles from baseline then omit dose for 24 h and rehydrate with 1 L NaCl 0.9% 8 hourly	Manage as for eGFR of 10–50 mL/min, but extend dose interval to 36 h	Prehydrate with 1 L NaCl 0.9% + 20 mmol KCL per 10 mL ampoule. Add slow Mag 2 tabs bd (535 mg tablets, 5.33 mmol Mg^2+^ Per tablet **+** slow K 2 tabs bd (600 mg tablet, 8 mmol K^+^ per tablet) If baseline renal impairment exists, aim is to rehydrate and attempt to restore normal eGFR while continuing fluconazole
Cotrimoxazole	75% of recommended dose for condition	25% of recommended dose for condition	Maintain fluid intake at > 1.5 L/day to prevent crystal formation Monitor K^+^ and glucose levels
Fluconazole	50% of recommended dose for condition	25% of recommended dose for condition	-
Kanamycin	12 mg/kg – 15 mg/kg three times per week	-	-
Rifafour/Rifinah	Rifampicin component at 10 mg/kg/day is considered safe in renal impairment	As for eGFR of 10 mL/min – 50 mL/min	Ethambutol-induced optic neuritis may occur; therefore, monthly eye examinations are recommended
TDF	Substitute with ABC/d4T/AZT as appropriate. If eGFR 30–50, then dose every 48 h if needed for hepatitis B co-infection	Do not use if eGFR < 30 mL/min or if the patient is on haemodialysis	Dose adjustments are possible when patients are co-infected with hepatitis B to prevent flares (see package insert for details) Increased monitoring

TDF, tenofovir disoproxil fumarate; eGFR, estimated glomerular filtration rate.

Tenofovir disoproxil fumarate may be continued in patients with chronic hepatitis B infection at a reduced dose (see package insert) because of the risk of a hepatic flare on withdrawal of the drug.^42^ However, if renal function declines on TDF or if there is severe dysfunction, then it is reasonable to replace TDF with an alternative NRTI and continue 3TC monotherapy, with or without pegylated interferon-α, for the management of hepatitis B.^42^ Assessment of hepatitis B surface antigen is therefore mandatory in all patients before TDF is discontinued and patients testing positive should be maintained on renally adjusted doses of TDF if possible (Table 4).

Reassuringly, patients who had their ART changed from TDF to an alternative NRTI because of nephrotoxicity showed high rates of recovery of renal function.^43^ Those who discontinued TDF were more likely to regain renal function compared to those who reinitiated TDF and there were no differences in virological outcomes between study groups.

### Patients initiated on tenofovir disoproxil fumarate with biochemical or clinical features of tenofovir disoproxil fumarate nephrotoxicity but preserved glomerular filtration rate

Subclinical tubular dysfunction is much more common than overt renal failure, but the long-term significance for kidney function and bone health is uncertain. In such cases, it may be reasonable to discontinue TDF and replace it with an alternative NRTI such as ABC. However, substituting TDF should be balanced not only against the increase in pill burden caused by providing an alternative ART regimen which is not available in a fixed, single dose combination, but also against the cost to the health sector. If a patient requires other potentially nephrotoxic medication such as aminoglycosides or amphotericin B, TDF should be replaced with ABC, AZT or d4T to avoid precipitating severe AKI. TDF can then be reinstituted on cessation of the nephrotoxic agent.^44^

## Conclusion

HIV-infected patients have a greatly increased risk of renal disease, a situation that is exacerbated by the frequent use of potentially nephrotoxic medication. Appropriate screening and monitoring of renal function in all HIV-infected patients is therefore essential. Considering that South Africa has limited capacity to care for patients with ESRD, managing other conditions which are known to cause AKI and CKD, such as hypertension and diabetes mellitus, is of paramount importance. Lack of integrated care and limited access to non-HIV-related medication in ART clinics result in many HIV-infected patients needing to attend multiple clinics, adding considerable cost and inconvenience to both patients and the health sector. Fragmented care also leads to duplication of tests, as well as the possibility of polypharmacy and prescription errors.

The lack of definitive care for patients with ESRD in South Africa means that only approximately 10% of those who would benefit from dialysis are receiving this therapy^47^ and as noted by Moosa et al.:^11^

The current stark reality in SA and many developing countries is that most people with ESKD [end-stage kidney disease] and HIV die as a result; some have limited access to dialysis. Most clinicians deal with advanced stages of CKD in HIV and prevention or early detection of renal disease in this population is neglected. Primary healthcare practitioners need a working system for screening, early detection and referral. (p. 7)

Renal disease is likely to continue to be a significant problem for South Africa’s HIV-infected population with implications for ART programme, as well as training of generalist healthcare workers regarding the management of renal disease.
